# Brain-clinical biotyping in patients with idiopathic REM sleep behavior disorder

**DOI:** 10.1038/s41531-025-01012-0

**Published:** 2025-06-07

**Authors:** Shi Tang, Bei Huang, Yanlin Wang, Yaping Liu, Jing Wang, Li Zhou, Siyi Gong, Yuhua Yang, Joey WY Chan, Steven WH Chau, Winnie CW Chu, Jill Abrigo, Jean-François Gagnon, Yun Kwok Wing

**Affiliations:** 1 Department of Psychiatry, Faculty of Medicine, The Chinese University of Hong Kong, Shatin, N.T., Hong Kong SAR China; 2 Li Chiu Kong Family Sleep Assessment Unit, Department of Psychiatry, Faculty of Medicine, The Chinese University of Hong Kong, Shatin, N.T., Hong Kong SAR China; 3 Li Ka Shing Institute of Health Sciences, Faculty of Medicine, The Chinese University of Hong Kong, Shatin, N.T., Hong Kong SAR China; 4 Brain and Mind Institute, The Chinese University of Hong Kong, Shatin, N.T., Hong Kong SAR China; 5 Center for Sleep and Circadian Medicine, The Affiliated Brain Hospital, Guangzhou Medical University, Guangzhou, Guangdong China; 6 Department of Neurology, Icahn School of Medicine at Mount Sinai, New York, NY USA; 7 Department of Imaging and Interventional Radiology, Faculty of Medicine, The Chinese University of Hong Kong, Shatin, N.T., Hong Kong SAR China; 8 Center for Advanced Research in Sleep Medicine, Hôpital du Sacré-Cœur de Montréal, Montreal, QC Canada; 9 Department of Psychology, Université du Québec à Montréal, Montreal, QC Canada

**Keywords:** Neuroscience, Neurology

## Abstract

Idiopathic REM sleep behavior disorder (iRBD) is a prodromal stage of α-synucleinopathies including Parkinson’s disease (PD), yet its clinical heterogeneity remains underexplored. This study aimed to identify novel brain-clinical biotypes in iRBD by integrating structural MRI and clinical assessments. We included 172 patients with video-polysomnography-confirmed iRBD and 126 controls who underwent multimodal MRI and clinical evaluation. Similarity Network Fusion was used to integrate cortical thickness, surface area, subcortical volume, and clinical data, followed by spectral clustering to identify iRBD biotypes. Two distinct biotypes were identified: Biotype 1 showed widespread cortical-subcortical-cerebellar atrophy, functional hypoconnectivity, more motor and cognitive deficits with higher prodromal PD risk; Biotype 2 demonstrated increased surface area in limbic and parietal regions, cortical-cerebellar hyperconnectivity, and preserved neurocognitive function. These findings underscore the presence of distinct neurobiological subtypes in iRBD, highlighting the need for longitudinal monitoring to clarify their trajectories and implications for disease progression.

## Introduction

Idiopathic/isolated REM sleep behavior disorder (iRBD) is a distinct parasomnia characterized by the enactment of dreams and excessive electromyographic activity during REM sleep (REM sleep without atonia, RSWA)^1^. Recent evidence indicates that iRBD serves as a specific prodromal stage of α-synucleinopathies, such as Parkinson’s disease (PD), dementia with Lewy bodies (DLB), and multiple system atrophy (MSA), with over 90% of iRBD patients converting to α-synucleinopathies within 15 years^2^. Therefore, recognizing iRBD and its associated neurodegenerative biomarkers is crucial for identifying the prodromal stage of α-synucleinopathies and predicting disease progression^3^. Several biomarkers have been identified in iRBD that may potentially forecast a faster disease progression, including autonomic dysfunction, olfactory loss, color vision impairment, neurocognitive impairment, dopamine dysfunction, excessive daytime sleepiness, depression, and motor function abnormalities^4^.

Meanwhile, increasing evidence underscores PD, DLB, and MSA as heterogeneous conditions, with potential subtypes exhibiting distinct clinical features and underlying mechanisms^5,6^. For example, previous endeavors have aimed at delineating different subtypes of PD and DLB via clinical^7,8^ and imaging biomarkers^9,10^. However, the heterogeneity of α-synucleinopathies might manifest not only at disease onset but also during its prodromal stage, characterized by the initial neuroanatomical spread of α-synuclein aggregates^11^. Thus, identifying different biotypes of iRBD will be helpful for facilitating understanding of iRBD heterogeneity and, as well as optimizing the efficacy of neuroprotective interventions.

A recent study has identified two subtypes of iRBD based on clinical symptoms, with subtype 1 characterized by higher non-motor symptom burden and widespread reduction of cortical gray matter volume than subtype 2^12^. However, the accuracy and reproducibility of these classification systems, grounded solely on clinical data, may have been limited^13^. An alternative and potentially more robust approach to categorizing patients with iRBD is to implement data integration techniques beyond using neuroimaging or behavioral/clinical features alone to identify brain-clinical biotypes^14,15^. This method offers the advantage of objectively capturing a diverse aspect of patient characteristics and exploring biological heterogeneity in vivo. By employing data-driven methods, researchers can achieve an unbiased detection of patient groups with similar brain-behavior profiles^13^.

In this study, we aim to integrate brain structural measures, including cortical thickness, surface area, and subcortical volume, and clinical assessments to identify subtypes in patients with iRBD, and to discover whether these biotypes will elucidate heterogeneities in brain structures and clinical characteristics in iRBD. To achieve this, we leveraged a novel multimodal data integration framework, similarity network fusion (SNF)^16^, which allows us to integrate structural brain features and clinical characteristics simultaneously in clustering, thus unveiling differential biotypes that are more readily interpretable in both clinical and neurobiological domains^17^. We hypothesized that there exist distinct iRBD biotypes that will be characterized by distinct neuroanatomic patterns and clinical profiles.

## Results

### Demographic characteristics of participants

In this study, a total of 300 subjects were initially recruited. Two subjects were excluded due to poor image quality, resulting in a final cohort of 172 patients with iRBD and 126 control subjects. There is no significant difference (*p* = 0.62) in age between the RBD group (mean age: 67.86 ± 6.94 years) and controls (mean age: 66.07 ± 6.92 years). Additionally, the RBD group exhibited a higher proportion (*p* < 0.05) of male participants (77.3%) compared to the control group (55.6%).

### Biotyping based on SNF

**Fig. 1:**
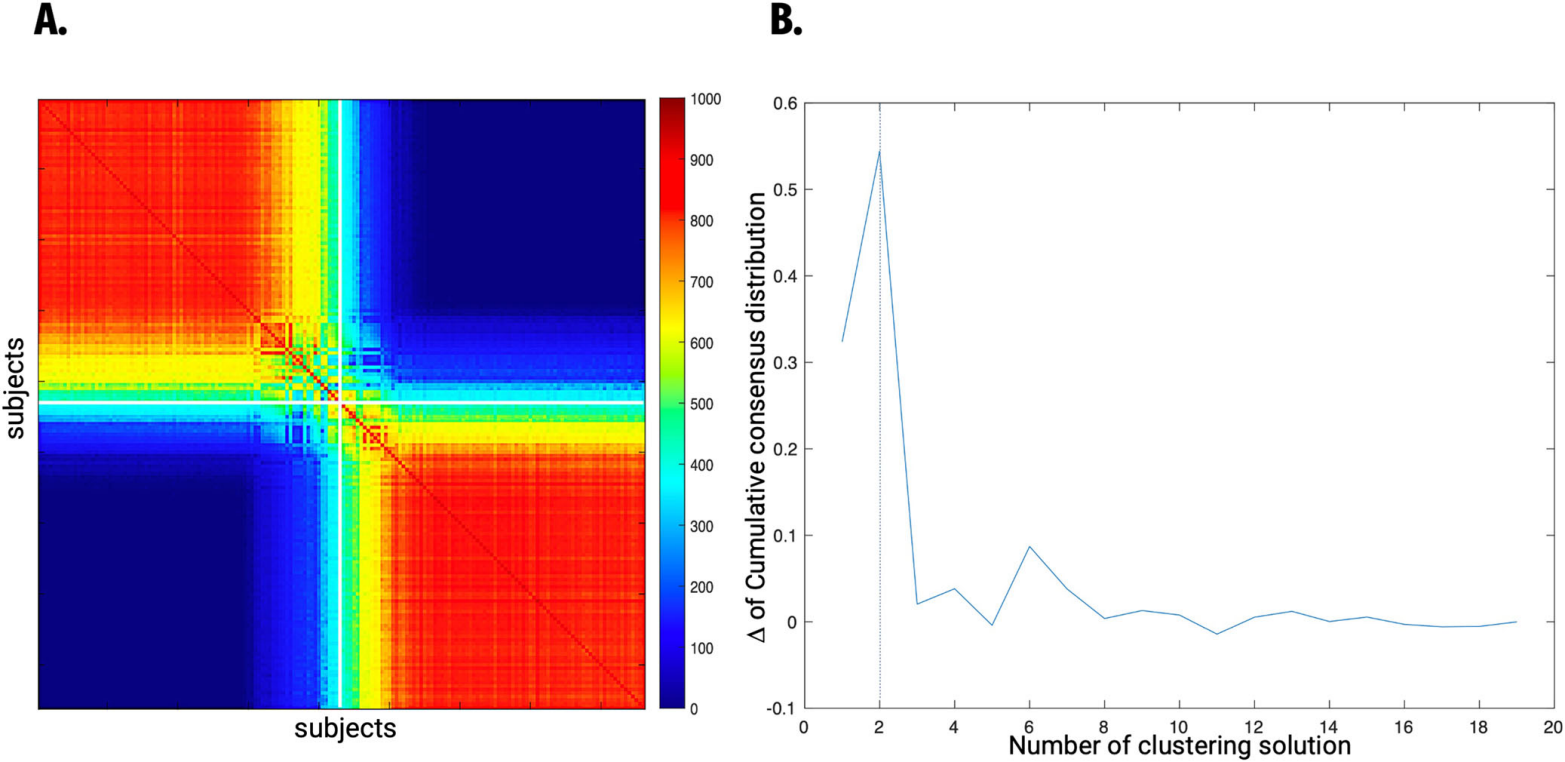
Finding a clustering solution based on consensus matrices. **A** consensus matrix of clustering results. 1000 iterations of bootstrapping (random sampling of 90% of participants) constructed a consensus matrix for a spectral clustering solution, which indicates how many times a pair of participants is clustered into the same group among 1000 iterations. **B** Determination of the optimal clustering solution. The optimal number of clusters was determined as ***C*** = 2, based on the largest Δ in the cumulative consensus distribution.

Based on the area under the curve for a cumulative distribution function (CDF) of consensus values (Fig. 1B), we focused on the subtyping results at clustering numbers *C* = 2, given that these Cs made the largest increases (ΔCDF > 0) of consensus degree. For this optimal solution, high stability of between-subject subgroups across bootstrapped samples was observed in the consensus matrices (Fig. 1A).

The number of participants classified into each of these two biotypes was Biotype 1: *n* = 86 and Biotype 2: *n* = 86. Tables 1 and S2 show the detailed demographic, clinical characteristics, and neurodegenerative markers of the two iRBD biotypes and controls. There was no significant difference in age, education, RBD onset age or symptom duration between the two biotypes. However, Biotype 1 had a significantly higher UPDRS-III score, lower HK-MoCA score, and higher prodromal PD risk score than Biotype 2, indicating more pronounced motor dysfunction, poorer cognitive functioning, and higher neurodegenerative risk in Biotype 1 relative to Biotype 2.

**Table 1:** Demographic and clinical characteristics of iRBD biotypes and controls

	Biotype 1^1^ (*n* = 86)	Biotype 2^2^ (*n* = 86)	Control^3^ (*n* = 126)	*p* value^&^	Post-hoc^@^
Age, year, mean (SD)	68.78 (6.77)	66.95 (7.03)	66.07 (6.92)	0.02	1 = 2, 1 > 3, 2 = 3
Sex, male (%)	70 (81.40)	63 (73.26)	70 (55.56)	<0.001	1 > 2 > 3
Education, above tertiary, *n* (%)	13 (15.12)	20 (23.26)	27 (21.43)	0.37	-
BMI, mean (SD)	24.85 (3.29)	24.55 (3.30)	24.67 (4.25)	0.86	-
Age of RBD symptoms onset, year, mean (SD)	60.17 (8.03)	59.49 (7.74)	-	0.14	-
RBD symptoms duration, years, mean (SD)	7.99 (5.67)	7.28 (5.35)	-	0.39	-
RBDQ-HK, total score, mean (SD)	38.73 (18.63)	38.29 (16.47)	9.57 (10.32)	<0.001	1 = 2 > 3
RBDQ-HK, factor 1 score, mean (SD)	13.32 (6.48)	13.64 (5.72)	6.03 (4.85)	<0.001	1 = 2 > 3
RBDQ-HK, factor 2 score, mean (SD)	25.41 (13.62)	24.64 (12.48)	3.55 (6.86)	<0.001	1 = 2 > 3
HK-MoCA, total score, mean (SD)	25.23 (2.66)	26.37 (2.91)	26.73 (2.06)	<0.001	1 < 2 = 3
UPDRS-III, total score, mean (SD)	8.27 (8.57)	4.36 (5.70)	1.67 (2.55)	<0.001	1 > 2 > 3
ESS, total score, mean (SD)	9.14 (5.32)	9.63 (5.28)	9.31 (5.38)	0.83	-
ISI, total score, mean (SD)	8.25 (5.30)	9.36 (5.34)	7.35 (5.62)	0.04	1 < 2, 1 = 3, 2 = 3
HADS, total score, mean (SD)	10.48 (6.71)	11.78 (7.10)	7.84 (5.79)	<0.001	1 = 2 > 3
SCOPA-AUT, total score, mean (SD)	13.84 (7.04)	12.48 (7.87)	9.00 (5.86)	<0.001	1 = 2 > 3
OIT, total correct score, mean (SD)	1.87 (1.82)	2.23 (2.00)	4.31 (1.66)	<0.001	1 = 2 < 3
Hue test, total error score, mean (SD)	192.53 (83.60)	163.38 (83.19)	153.58 (88.29)	0.006	1 = 2, 1 > 3, 2 = 3
Total estimated LR for prodromal PD with log transformation, mean (SD)	3.52 (0.96)	2.97 (1.07)	0.32 (0.39)	<0.001	1 > 2 > 3

^&^*p* value was calculated with one-way ANOVA, ANCOVA, or chi-square test.

^@^Bonferroni correction was applied to adjust for multiple comparisons of three groups, significance was thresholded at *p* < 0.017.

*iRBD* isolated Rapid eye movement sleep behavior disorder; *BMI* Body mass index, *RBDQ-HK* RBD questionnaire-Hong Kong, *MoCA* Montreal Cognitive Assessment, *UPDRS* Unified Parkinson’s Disease Rating Scale, *ESS* Excessive Daytime Sleepiness, *ISI* Insomnia Severity Index, HADS Hospital Anxiety and Depression Scale, *SCOPA-AUT* Scales for Outcomes in Parkinson’s Disease-Autonomic, *OIT* Olfactory Identification Test, *MDS* Movement Disorder Society, *LR* Likelihood ratio, *PD* Parkinson’s disease.

### Neuroanatomic pattern of the 2 iRBD biotypes

Compared to controls, iRBD patients from both biotypes showed no significant differences in cortical thickness. Additionally, no significant differences were observed between Biotype 1 and Biotype 2 (Table S3).

**Fig. 2:**
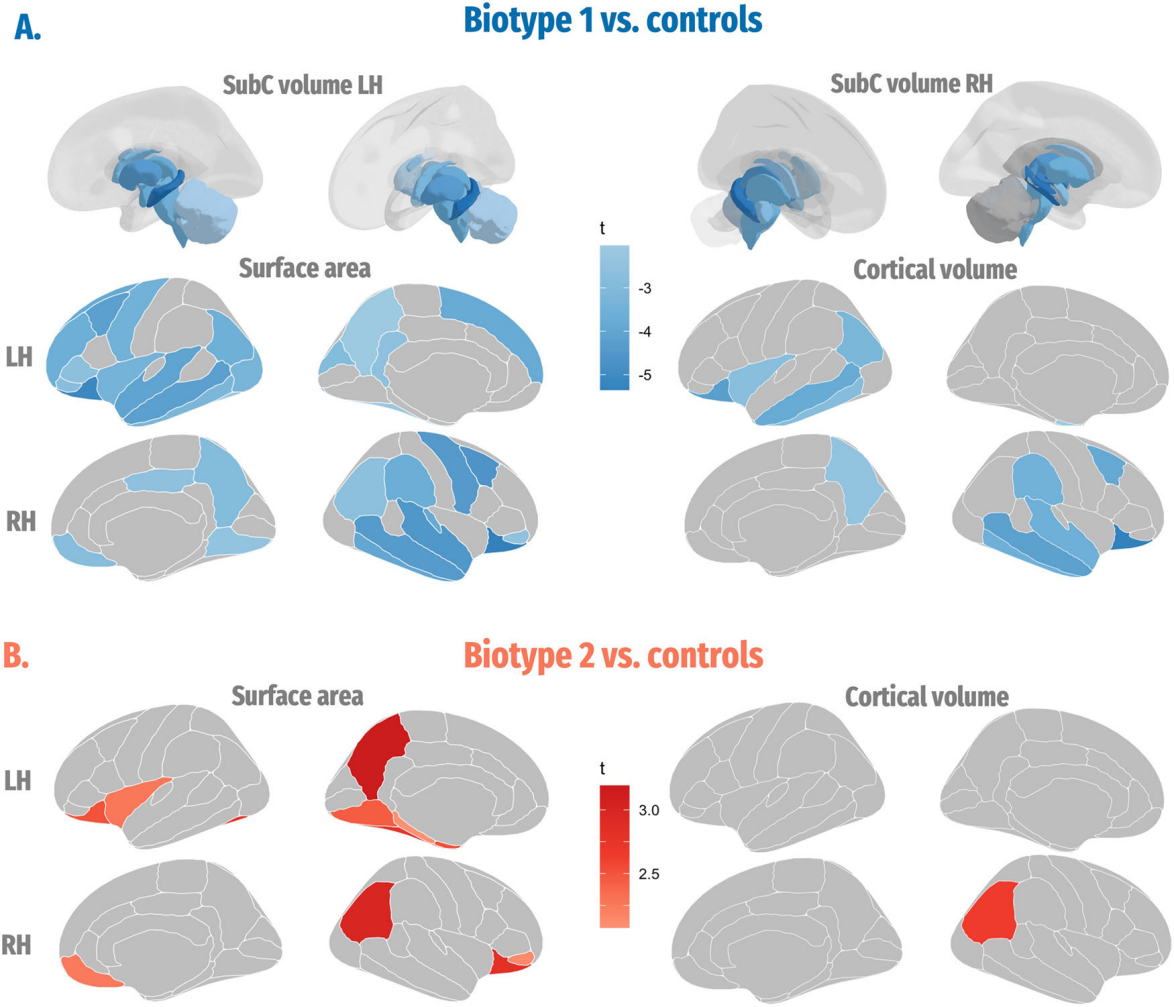
Brain structure differences between each iRBD biotype and the control group. **A** Group difference in subcortical volume, cortical surface area and cortical volume between Biotype 1 patients and controls. **B** Group difference in cortical surface area and cortical volume between Biotype 2 patients and controls. The color bar represents t-statistics, with red indicating increase and blue indicating decrease. L/RH, left/right hemisphere.

In terms of cortical surface area, Biotype 1 patients demonstrated significantly smaller surface areas in 17 of 34 left-hemisphere regions of interest (ROIs) and 14 of 34 right-hemisphere ROIs, spanning frontal, temporal, and occipital regions. Conversely, Biotype 2 patients exhibited significantly larger surface areas in 7 of 34 left-hemisphere ROIs and 4 of 34 right-hemisphere ROIs, including limbic and occipital regions (Fig. 2 and Table S4). Additionally, Biotype 1 patients demonstrated more extensive surface area reductions than Biotype 2 patients across the limbic, frontal, temporal, and occipital regions (Fig. S1 and Table S4).

Cortical and subcortical volume analyses revealed that Biotype 1 patients had significantly smaller volumes in 19 of 24 subcortical ROIs, whereas no differences in subcortical volume were observed in Biotype 2 patients. Additionally, patients with Biotype 1 exhibited significantly smaller cortical volumes in 5 of 34 left-hemisphere ROIs and 8 of 34 right-hemisphere ROIs, encompassing frontal, temporal, and parietal regions relative to controls. In contrast, Biotype 2 patients demonstrated increased volume solely in the right inferior parietal cortex (Fig. 2 and Table S5–S6). Furthermore, Biotype 1 patients showed more extensive volume reductions than Biotype 2 patients across the subcortical nuclei, as well as the limbic, frontal, temporal, and occipital regions (Fig. S1 and Table S5–S6).

**Fig. 3:**
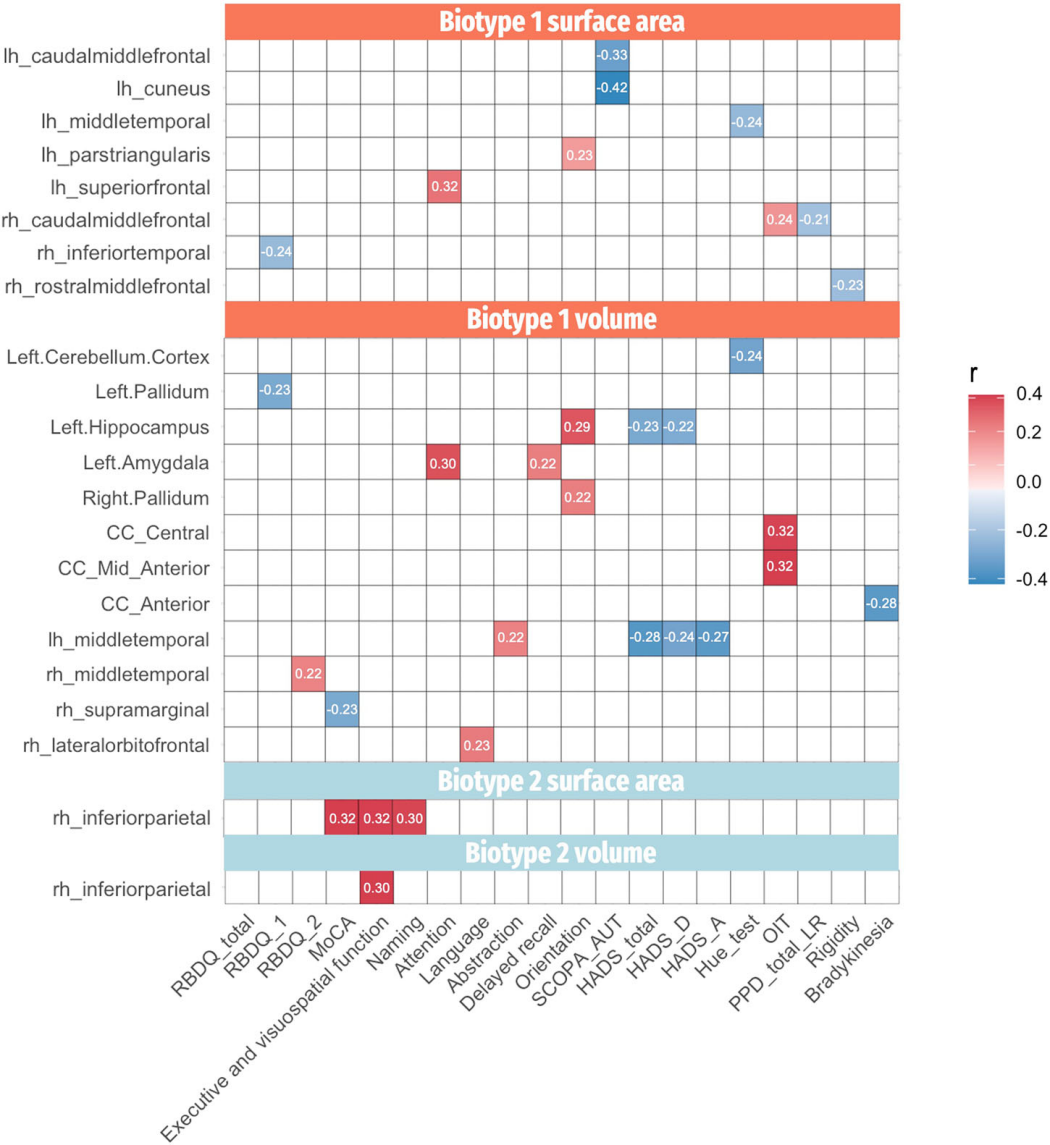
Correlations between neuroanatomic matrices and clinical profiles for each biotype. Only significant results are displayed, with the color indicating the corresponding r-values. lh left hemisphere, rh right hemisphere, CC cingulate cortex. RBDQ RBD questionnaire, MoCA Montreal Cognitive Assessment, SCOPA-AUT Scales for Outcomes in Parkinson’s Disease-Autonomic, HADS Hospital Anxiety and Depression Scale, OIT Olfactory Identification Test, PPD prodromal Parkinson’s disease, LR Likelihood ratio.

All the above results survived further Bonferroni multiple testing correction. In patients with Biotype 1, the relationship between cortical/subcortical matrices and clinical symptoms is intricate. The interplay is highlighted by the association of decreased surface area and volume with various clinical scores, indicating a multifaceted impact on symptomatology (Fig. 3). While Biotype 2 patients exhibit a clearer trend, as increased surface area and volume were mainly associated with better cognitive function (Fig. 3).

### Functional connectivity of the 2 biotypes

**Fig. 4:**
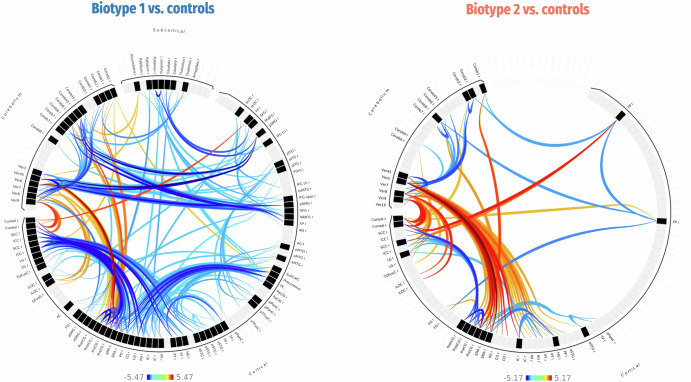
Functional connectivity differences between each iRBD biotype and the control group. Only significant connections are displayed (thresholded at connection level *p* < 0.001 and multiple-comparison corrected using *p*-FDR < 0.05). The color bar represents t-statistics, with warm colors indicating hyperconnectivity and cold colors indicating hypoconnectivity.

When compared to controls, iRBD patients with Biotype 1 predominantly exhibited extensive cortical-subcortical-cerebellar hypoconnectivity, along with hyperconnectivity between the cerebellum and sensorimotor cortex. In contrast, Biotype 2 patients primarily demonstrated cerebellar-cortical hyperconnectivity, accompanied by scattered cortical-cerebellar hypoconnectivity (Fig. 4). Additionally, Biotype 1 patients also demonstrated pronounced cortical-subcortical-cerebellar hypoconnectivity compared to Biotype 2 patients (Fig. S1). Furthermore, the additional supplementary FC analysis using the DK cortical and aseg subcortical atlases reaffirmed the original findings of Biotype 1, demonstrating widespread cortical-subcortical hypoconnectivity (Fig. S2). However, no statistically significant FC changes were observed in Biotype 2.

## Discussion

In this study, we identified two distinct biotypes in patients with iRBD through a comprehensive SNF approach that integrated multiple structural imaging phenotypes and clinical measures. Our findings were supportive of two distinct neuroanatomic phenotypes within iRBD patients, each exhibiting unique characteristics compared to control subjects. Notably, patients classified under Biotype 1 displayed cortical and subcortical atrophy and cortical-subcortical-cerebellar hypoconnectivity, while those in Biotype 2 exhibited relative cortical volume increase and mainly cortical-cerebellar hyperconnectivity. The most notable discrepancies in symptomologies between the two biotypes were the significantly more prominent motor dysfunction and lower cognitive functioning in Biotype 1.

Subcortical volume reductions are also observed in Biotype 1 patients with iRBD compared to controls. Consistent findings across previous studies highlight reduced volumes in key subcortical regions such as the putamen, pallidum, and caudate in individuals with iRBD^18–20^. These findings correspond with observations of presynaptic dopaminergic dysfunction in dopamine transporter single-photon emission computed tomography studies^21^. Brainstem volume reduction has been identified, suggesting an underlying dysfunction in the nuclei that modulate REM sleep muscle tone^22^. Furthermore, patients in Biotype 1 also exhibit cortical atrophy, evidenced by decreased surface area and volume reduction across various cortical regions, especially limbic, temporal, and parietal areas. Previous studies support these findings, showing volume decreases in frontal^20,23,24^, cingulate^23,25^, occipital and parietal^20^, and temporal regions^20,25^ in patients with iRBD. Cortical atrophy likely reflects a loss of neuronal integrity, which is typical of neurodegeneration.

On the other hand, our study found that Biotype 2 patients did not have significant differences in subcortical volume compared to controls. Furthermore, Biotype 2 patients show cortical volume increase, with increased surface area in limbic regions and volume in the right inferior parietal gyrus. A multicenter study previously identified increased surface area in the inferior temporal and parahippocampal regions in iRBD patient population^26^, albeit there was no further elaboration on this intriguing phenomenon. Strictly speaking, cortical volume increase does not reflect neurogenesis, a rare event in adult brains outside specific areas like the hippocampus, subventricular zone, and olfactory bulb, but it is more likely part of a complex reactive process^27^. Increased gray matter volume may represent adaptive plasticity, such as cerebral edema, reactive gliosis, and increased neuronal branching, serving to temporarily compensate or mask underlying pathology^28^. Some studies discussed brain compensation during the early stages of neurodegenerative conditions like Alzheimer’s disease (AD) and Huntington’s disease^29–31^, such that a greater gray matter volume was protective against clinical deterioration, supporting the role of brain reserve. Prior research also highlights the association between brain volume and functional reserve markers in PD and other neurodegenerative diseases^32^. The positive association between increased surface area and cognitive functioning in Biotype 2 also suggests that structural variations may aid functional maintenance despite underlying pathology. Thus, enhancing brain reserve might potentially alleviate symptoms and slow the progression of synucleinopathies. The neuronal mechanisms underlying potential compensation are complex, necessitating further longitudinal data and intervention studies to validate the compensation hypothesis^29^.

In this study, no significant differences in cortical thickness were observed between the two biotypes, as well as between iRBD and the control group. It is proposed that the mechanisms governing the horizontal and vertical development of the cerebral cortex might operate independently, influencing cortical surface area and thickness separately^33^. Gray matter volume is a composite of these indices, thus reflecting both traits^34^. Previous research indicated cortical thinning in several regions among patients with iRBD compared to healthy controls, such as the lingual gyrus^18^, fusiform gyrus^18,24^, lateral occipital cortex^18,35^, orbitofrontal, dorsolateral frontal, and cingulate cortex^19^. Cortical and subcortical atrophy in iRBD is more severe and extensive in patients who also have mild cognitive impairment^36,37^. Though reduced surface area is suggested to be a major factor contributing to volume reduction^38^, few studies have investigated surface area changes in individuals with iRBD or cognitive decline^39^. Future longitudinal studies are warranted to explore whether surface area alterations precede cortical thickness changes in RBD.

Both biotypes exhibit mixed patterns of functional connectivity alterations compared to controls. Biotype 1 patients primarily demonstrate widespread cortical-subcortical-cerebellar hypoconnectivity with limited cerebellar-sensorimotor hyperconnectivity. In contrast, Biotype 2 patients display cortical-cerebellar hyperconnectivity with minimal cortical and cerebellar hypoconnectivity. Similarly, distinct neurodegenerative diseases manifest different FC patterns. For instance, AD consistently exhibits reduced connectivity in the default mode network, correlating with dementia severity^40^. Our previous meta-analysis of FC studies in α-synucleinopathies revealed widespread abnormal crosstalk across brain networks with both hypo- and hyperconnectivity^41^. Hypoconnectivity, indicative of internetwork disruption, may underlie functional disconnection and pathology spread across interconnected networks. Hyperconnectivity (that is, increased synchrony of spontaneous BOLD fluctuations) is also a common finding in resting-state fMRI studies across a range of neurodegenerative diseases^42^. The exact mechanism of hyperconnectivity remains unclear, with some activation of existing network regions was proposed as a compensatory strategy. Notably, both biotypes exhibit some possibly functional compensation, with Biotype 2 showing predominance. This aligns with the scaffolding theory of aging, suggesting increased activation as an adaptive response using compensatory scaffolding to counteract neural decline by utilizing alternative neural circuits for cognitive function maintenance^43^. Other compensation models propose augmented activation within existing networks as contributory factors^44^.

Pathologically, DLB and PD are thought to arise from the propagation and accumulation of misfolded α-synuclein in the brain^45^. Braak et al.‘s hypothesis suggests that pathological α-synuclein aggregates and dysfunction initiate in the lower brainstem and ascend to the midbrain and cerebral cortex, potentially leading to iRBD and subsequent phenoconversion to PD or DLB^46,47^. While there are two proposed subtypes of α-synuclein pathology progression in PD: “brain-first” (starting in the brain and spreading to the peripheral system) and “body-first” (originating in the enteric or peripheral autonomic nervous system and spreading to the brain), with iRBD being suggested as an early indicator of the “body-first” subtype^48^. In addition, an emerging concept redefines PD and DLB from a biological definition based on the detection of pathological α-synuclein^49^. Our findings introduce a novel biotyping framework for iRBD based on neuroimaging-clinical integration via SNF analysis. Specifically, Biotype 1 aligns with typical neurodegeneration patterns, while Biotype 2 exhibits an ‘atypical’ pattern characterized by relative brain volume preservation or even increases, suggestive of a potential compensatory reserve. The observed gradient in prodromal PD risk (Biotype 1 > Biotype 2 > Controls) further supports the validity of this biotypic stratification and underscores its potential for refining prognostic models. These findings also raise an important question: Could distinct biotypes reflect differential responses to α-synuclein pathology or represent divergent disease progression phenotypes? Addressing this question through longitudinal studies will be essential to further elucidate the clinical and biological significance of these biotypes.

This study has some limitations. First, the absence of a validation dataset underscores the need for future research with larger cohorts to validate current findings. Second, the cross-sectional study inherently limits our ability to predict the phenoconversion to Parkinsonism-first or dementia-first trajectories, so longitudinal studies are warranted to address this gap^50^. In addition, not all participants were definitively clustered into specific biotypes, which is reasonable given the inherent complexity of the population under study. Therefore, the identified biotypes may serve as an initial step in addressing disease heterogeneity, based on neuroanatomic and clinical characteristics. Future investigations should expand input features to include clinical, genetic, environmental, and additional neuroimaging factors for a more comprehensive understanding. Extensive research on clinical heterogeneity, coupled with advances in biological markers, will guide the path towards precision medicine, enabling targeted and effective interventions.

In conclusion, we identified two distinct biotypes of iRBD, each with unique clinical, neuroanatomic, and functional connectivity features, which suggest distinct neurobiological subtypes in iRBD. Longitudinal monitoring of these biotypes and integrating with other omics data will be essential to elucidate their trajectories and implications for disease progression. Our biotyping framework provides a testable model for future research to investigate differential progression trajectories and potentially inform more targeted therapeutic development for iRBD patients.

## Methods

### Study design and participants

This is a cross-sectional study employing data-driven clustering to identify biotypes within an iRBD cohort, with secondary comparisons to controls. Patients with iRBD were recruited from our ongoing RBD cohort in the Li Chiu Kong Family Sleep Assessment Unit at Shatin Hospital, Shatin, Hong Kong^51–53^, from September 2021 to November 2023. Control participants, comprised of neurologically healthy individuals, were recruited from both local community and the Sleep Assessment Unit.

Diagnosis of iRBD among patients was confirmed via video-polysomnography assessment, adhering to the diagnostic criteria of the International Classification of Sleep Disorders, 3rd edition^1^. The exclusion criteria for all subjects included: RBD features related to narcolepsy and other pre-existing neurodegenerative disorders; and presence of MRI contraindications (e.g., metal implants or claustrophobia). Ethical approval was obtained from the Clinical Research Ethics Committee of the Joint Chinese University of Hong Kong and New Territories East Cluster, with a reference number of 2021.277. All individuals provided written informed consent.

### Questionnaires and clinical assessments

A comprehensive questionnaire was administered to all participants.

1. General questionnaire: A structured questionnaire captured socio-demographic information, lifestyle habits (e.g., tea, coffee, alcohol consumption, smoking status), sleep-wake patterns, and common sleep symptoms such as insomnia.
2. Excessive daytime sleepiness: The Epworth Sleepiness Scale (ESS) was utilized to quantify excessive daytime sleepiness.
3. Insomnia severity: Severity of insomnia symptoms was assessed using the Insomnia Severity Index (ISI).
4. Anxiety and depression symptoms: The Hospital Anxiety and Depression Scale (HADS) was employed to evaluate the severity of depressive and anxiety symptoms.
5. Autonomic dysfunction symptoms: Symptoms such as constipation, erectile dysfunction, urinary dysfunction, and symptomatic hypotension were evaluated using the Scales for Outcomes in Parkinson’s Disease-Autonomic (SCOPA-AUT).
6. Severity of RBD symptoms: the clinical aspects and severity of RBD features were assessed by RBD questionnaire–Hong Kong (RBDQ-HK), a locally designed and validated questionnaire comprising 13 questions^54^. It contains two components: (1) dream-related factors (factor 1) and (2) behavioral factors (factor 2).

In addition, participants underwent a series of clinical, neuropsychiatric, neurocognitive, and autonomic function assessments:

1. Motor function: The Unified Parkinson’s Disease Rating Scale part III (UPDRS-III)^55^ measured motor function.
2. Cognitive function: The Hong Kong version of the Montreal Cognitive Assessment (HK-MoCA)^56^ was used to assess global cognitive function.
3. Psychiatric disorders: Lifetime psychiatric disorders were evaluated via structured interviews based on DSM-IV criteria and supplemented by clinical records.
4. Sleep disorders: Diagnostic Interview for Sleep Patterns and Disorders (DISP)^57^, a structured diagnostic interview, was used to further evaluate RBD symptoms, sleep patterns, and other sleep disorders.
5. Olfactory function: The Olfactory Identification Test (OIT)^58^ was used to assess olfactory function.
6. Color vision discrimination: The Farmsworth-Munsell 100 hue test was conducted to assess color vision discrimination^59^.
7. Prodromal PD likelihood: The 2019 version of Movement Disorder Society (MDS) criteria for prodromal PD^60^ was used to determine the overall likelihood ratio (LR) for prodromal PD, with the likelihood ratio being transformed using Log base 10.

### MRI acquisition and processing

MRI examinations were performed using a whole-body 3.0 T MRI scanner (MAGNETOM Prisma; Siemens AG, Munich, Germany) equipped with a 64-channel head coil at the Prince of Wales Hospital, Hong Kong. Participants were comfortably positioned in the coil and provided with soft earplugs to mitigate external noise interference. Additional stabilization of the head was ensured using foam pads to minimize potential motion artifacts of the participant. High-resolution sagittal T1-weighted anatomical images were acquired utilizing a sagittal 3D T1-weighted Magnetization Prepared Rapid Gradient Echo (MPRAGE) sequence with the following parameters: repetition time (TR) = 2300 ms, echo time (TE) = 3.06 ms, flip angle = 9°, and voxel size = 1 mm³ isotropic. Resting-state functional MRI (rsfMRI) images were obtained using a Gradient-Recalled Echo-Planar Imaging pulse sequence with TR = 1500 ms, TE = 30 ms, flip angle = 70°, and voxel size = 2 × 2 × 3 mm³. During rsfMRI scanning, participants were instructed to relax with their eyes closed, ensuring that they remained awake and motionless throughout the procedure.

T1-weighted images were processed using FreeSurfer software (version 7.1.0) on the same workstation, employing its fully automatic pipeline^61^. For each subject, FreeSurfer parcellated 34 cortical ROIs per hemisphere based on the Desikan-Killiany atlas^62^ and 24 subcortical ROIs. Regional cortical thickness, cortical surface area, and cortical/subcortical volume were extracted.

Resting-state fMRI data analyses were conducted using Statistical Parametric Mapping (SPM, https://www.fil.ion.ucl.ac.uk/spm/) version 12 and the CONN toolbox^63^. These software packages offer methods to mitigate head motion artifacts and accurately identify correlated and anti-correlated networks. To address potential spurious correlations in resting-state networks, preprocessing and cleaning of the fMRI signal were performed. The Artifact Detection Toolbox in CONN was employed to identify problematic time points characterized by head displacement exceeding 0.5 mm or global mean intensity deviating more than 3 standard deviations from the mean image intensity. Runs with over 30% outlier images were excluded from analysis, although no participants were excluded due to excessive motion. The first-level general linear model (GLM) incorporated participant motion parameters, including rotation and translation parameters and their derivatives, as well as artifactual covariates. Anatomical volumes were segmented into gray matter, white matter, and cerebrospinal fluid areas, with resulting masks eroded to minimize partial volume effects. Noise-signal for anatomical CompCor correction was extracted from the union of eroded white matter and ventricle masks, transformed to native functional space. Six CompCor parameters, Friston-24 motion parameters, and linear trend were regressed from the timeseries data using a GLM. The resulting residual blood-oxygen-level-dependent (BOLD) timeseries underwent band-pass filtering (0.008 Hz < f < 0.09 Hz). We predefined 132 ROIs in the CONN toolbox based on the Harvard–Oxford cortical & subcortical structural atlases (https://neurovault.org/collections/262/) and AAL atlas cerebellar areas^64^. Additionally, we conducted a supplementary functional connectivity analysis using the same atlas for the structural analyses, i.e., Desikan-Killiany cortical atlas and the aseg subcortical atlas.

Pearson’s correlation coefficients were calculated to determine the correlation between the timeseries of each pair of regions. Subsequently, Fisher’s r-to-z transformation was applied to correlation coefficients.

### Similarity network fusion and spectral clustering

Biotyping was performed using similarity network fusion (SNF), a method for integrating multiple data sources into one comprehensive graph^16^. This graph captures the relationship between samples by combining independent similarity networks generated from each data source using a K-nearest neighbors weighted kernel. Through an iterative process of combining these networks via a non-linear message passing protocol, SNF creates a final network that incorporates information from all data sources. This integrated network can then be used for various analytical techniques, such as clustering or graph analysis, for further exploration and interpretation. SNF has demonstrated effectiveness in unraveling heterogeneity within neuropsychiatric populations^14,65^. SNF was used to integrate structural imaging (cortical thickness, surface area, and subcortical volume) and clinical data types (see Table S1 listing all SNF input features). The SNF algorithm was conducted in Matlab_R2023a, which has been previously described^16^ (http://compbio.cs.toronto.edu/SNF/).

Before applying SNF, imaging features were preprocessed by regressing out age, sex, and handedness (and total intracranial volume for surface area and volume) to account for potential confounders. Specifically, for each imaging feature Y (e.g., volume and surface area), the following regression model was applied:

$${\rm{Y}}={\rm{\beta }}0+{\rm{\beta }}1\cdot {\rm{age}}+{\rm{\beta }}2\cdot {\rm{sex}}+{\rm{\beta }}3\cdot {\rm{handedness}}+{\rm{\beta }}4\cdot {\rm{TIV}}+{\rm{\epsilon }}$$

The adjusted feature values used in subsequent analyses were the residuals (*ϵ*) from this model. Additionally, each input feature for SNF was normalized using z-scoring. Biotyping using SNF involved the following steps:

1. Calculation of a sample similarity network for each data type: Four between-subjects distance matrices were computed for cortical thickness, surface area, subcortical volume, and clinical symptoms separately. The distance matrix was calculated consistently using the ‘cosine similarity’ kernel to generate between-subjects affinity matrices, with each cell representing the similarity of distance profiles between two participants. The scaled exponential similarity kernel was used for this calculation.
2. Integration of these networks into a SNF-combined similarity network: The resulting four affinity matrices were input to SNF to create a single fused affinity matrix. Parameters such as the number of nearest neighbors (*k*), the number of iterations (*T*), and hyperparameters related to feature scaling (*μ*) were set as recommended in the original paper (*k* = 30, *T* = 20, *μ* = 0.5)^16^ to ensure reproducibility of biotype findings.
3. Identification of homogeneous biotypes: Spectral clustering was used to identify biotypes by inputting the single fused affinity matrix into a spectral clustering algorithm. Following the methodology outlined in the original work of SNF^16^, we utilized a traditional spectral method that combines MinCut and equipartitioning to minimize the objective function using a normalized Laplacian matrix^66^. To ensure more reproducible and outlier-robust biotype findings, we iteratively performed the SNF process 1000 times based on bootstrapped samples (resampling 90% of cases without replacement) and created a consensus matrix with varying clustering numbers (C) from 2 to 20. Each cell in the consensus matrix indicates the consistency of grouping a pair of participants together across 1000 iterations at a specific clustering number (2–20).
4. Clustering solution evaluation: The SNF process was iterated 1000 times based on bootstrapped samples to construct a consensus matrix. By varying the clustering number (C) from 2 to 20, the degree of consensus for each clustering solution was evaluated using the cumulative consensus distribution approach. The clustering solution with the highest stability across different sampled cases was selected.

**Fig. 5:**
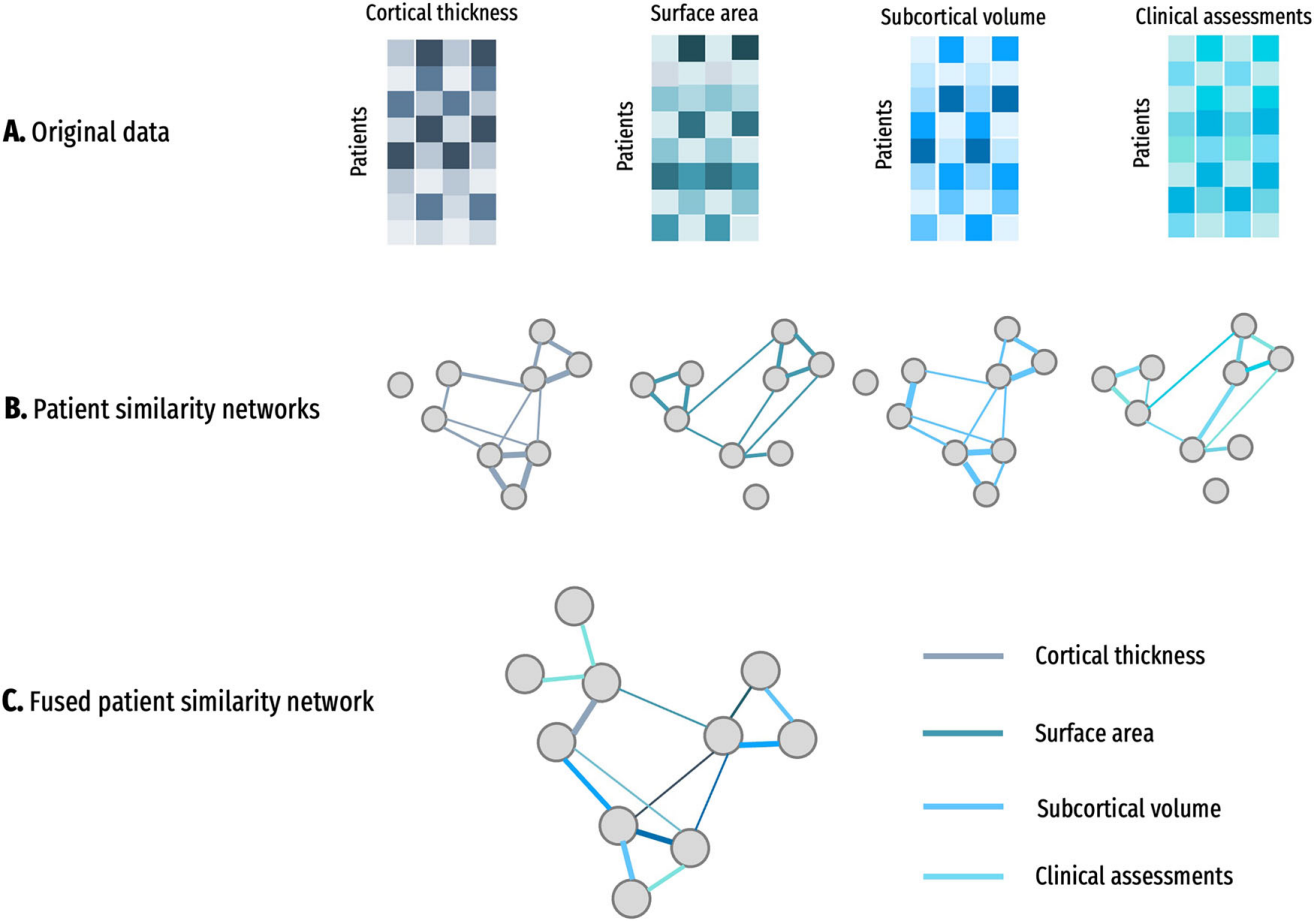
Illustrative example of SNF steps. **A** Example representation of cortical thickness, surface area, subcortical volume, and clinical datasets for the same cohort of patients. **B** Patient-by-patient similarity networks, equivalent to the patient-by-patient data. Patients are represented by nodes, and patients’ pairwise similarities are represented by edges. **C** The iterative network fusion results in convergence to the final fused network. Edge color indicates which data type has contributed to the given similarity.

The illustrative example of SNF steps was shown in Fig. 5.

### Statistical analysis

All acquired demographic and clinical data were processed and analyzed using standard statistical analysis methods with SPSS 27.0 (Chicago, IL, USA) and R project Version 4.3.2. Descriptive data will be presented as means ± SD or frequencies (%). Categorical data will be analyzed using chi-square or Fisher’s exact probability test. Continuous data will be compared among groups using Analysis of covariance (ANCOVA; age and sex as covariates) and Kruskal-Wallis tests. For post-hoc analysis, the Bonferroni test will be used to account for the multiple comparison effect. A *p* < 0.05 is considered statistically significant for all analyses.

To illustrate the neuroanatomic pattern in different biotypes, the cortical thickness, surface area, cortical/subcortical volume were compared among the 2 biotypes and controls using ANCOVA tests (age, sex, and handedness as covariates for all measurements and total intracranial volume as extra covariate for surface area and volume measurement). We applied region-specific corrections based on the number of comparisons within each anatomical domain^67^. For cortical measurements, a *p* ≤ 0.001 is considered statistical significance to correct multiple comparisons of 34 regions on each hemisphere, aligns with Bonferroni correction for 34 comparisons (0.05/34 ≈ 0.001). For subcortical volume, a *p* < 0.005 is considered statistical significance to correct multiple comparisons of 10 subcortical regions (0.05/10 = 0.005) on each hemisphere. For post-hoc between-group analysis, the Bonferroni test will be used to account for the multiple comparison effect. Functional connectivity analyses were thresholded at a connection-level *p* < 0.001, with multiple comparisons corrected by false discovery rate at *p* < 0.05.

Pearson correlation or Spearman rank correlation analyses were conducted separately for each biotype to identify neuroanatomic alterations (regions with significant changes in biotype vs. controls) that were significantly correlated (*p* < 0.05) with clinical symptomatology. The imaging features consisted of aforementioned processed data from regions showing significant differences between biotypes and controls. Clinical scores encompassed various measures, including RBDQ-HK (factor 1, factor 2, and total scores), UPDRS-III total score, HK-MoCA total score, HADS (depression, anxiety, and total scores), SCOPA-AUT total score, Color Hue Test score, OIT score, and MDS- prodromal PD total LR.

## Supplementary information

Supplementary materials-brain biotyping-RBD.

STROBE-checklist-biotyping RBD

## Data Availability

The data that support the findings of this study are available from the corresponding author, upon reasonable request.

## Abbreviations

AD: Alzeimer’s disease
ANCOVA: Analysis of covariance
BOLD: Blood-oxygen-level-dependent
CDF: Cumulative distribution function
DISP: Diagnostic Interview for Sleep Patterns and Disorders
DLB: Dementia with Lewy bodies
DSM-IV: Diagnostic and Statistical Manual of Mental Disorders, fourth edition
ESS: Epworth Sleepiness Scale
FC: Functional connectivity
FDR: False discovery rate
GLM: General linear model
HADS: Hospital Anxiety and Depression Scale
HK-MoCA: The Hong Kong version of the Montreal Cognitive Assessment
iRBD: idiopathic/isolated REM sleep behavior disorder
ISI: Insomnia Severity Index
LR: Likelihood ratio
MDS: Movement Disorder Criteria
MPRAGE: Magnetization Prepared Rapid Gradient Echo
MRI: Magnetic Resonance Imaging
MSA: Multiple system atrophy
OIT: Olfactory Identification Test
PD: Parkinson’s disease
RBDQ-HK: RBD questionnaire-Hong Kong
ROIs: Regions of interest
rsfMRI: Resting-state functional MRI
RSWA: REM sleep without atonia
SNF: Similarity network fusion
SCOPA-AUT: Scales for Outcomes in Parkinson’s Disease-Autonomic
SPM: Statistical Parametric Mapping
TIV: Total intracranial volume
TR: Repetition time
TE: Echo time
UPDRS-III: The Unified Parkinson’s Disease Rating Scale part III
v-PSG: video-Polysomnography
