# Supplementary material for: Brain-clinical biotyping in patients with idiopathic REM sleep behavior disorder
Source: NPJ Parkinsons Dis. 2025 Jun 7;11:156. doi: 10.1038/s41531-025-01012-0 (PMC12145420; doi:10.1038/s41531-025-01012-0)
Supplement: Supplementary file 1 — Supplementary materials-brain biotyping-RBD. [file 41531_2025_1012_MOESM1_ESM.pdf]

Supplementary Materials

Brain-clinical biotyping in patients with idiopathic REM sleep behavior disorder

Table S1. Model variables by data type.

Table S2. Clinical and neurogenerative markers of iRBD biotypes and controls.

Table S3. Cortical thickness of cortical regions in Biotype 1, Biotype 2 and control.

Table S4. Surface area of cortical regions in Biotype1, Biotype 2 and control.

Table S5. Volume of cortical regions in Biotype1, Biotype 2 and control.

Table S6. Subcortical volume (mean, mm^3) in Biotype 1, Biotype 2 and control.

Fig. S1. Group differences of cortical surface area, volume, subcortical volume, and functional connectivity between Biotype 1 and Biotype 2 patients.

Fig. S2. Functional connectivity differences between Biotype 1 and the control group using Desikan-Killiany and aseg atlas.

Table S1. Model variables by data type.

Data Type	Variables
**Cortical Thickness**	Thickness (mm) measures (bilateral):<br>- Banks of the superior temporal sulcus<br>- Cuneus<br>- Entorhinal<br>- Frontal pole<br>- Fusiform gyrus<br>- Inferior and superior parietal gyrus<br>- Inferior and superior temporal gyrus<br>- Insula<br>- Lateral occipital gyrus<br>- Lateral and medial orbitofrontal gyrus<br>- Lingual gyrus<br>- Paracentral gyrus<br>- Parahippocampal gyrus<br>- Pars opercularis, pars triangularis, and pars orbitalis<br>- Pericalcarine<br>- Postcentral gyrus<br>- Precentral gyrus<br>- Precuneus<br>- Rostral and caudal middle frontal gyrus<br>- Rostral anterior, caudal anterior, posterior and isthmus of the cingulate<br>- Superior, middle and inferior temporal gyrus<br>- Supramarginal gyrus<br>- Temporal pole<br>- Transverse temporal gyrus
**Surface area**	Area (mm^2) measures (bilateral):<br>- Banks of the superior temporal sulcus<br>- Cuneus<br>- Entorhinal<br>- Frontal pole<br>- Fusiform gyrus<br>- Inferior and superior parietal gyrus<br>- Inferior and superior temporal gyrus<br>- Insula<br>- Lateral occipital gyrus<br>- Lateral and medial orbitofrontal gyrus<br>- Lingual gyrus<br>- Paracentral gyrus

	- Parahippocampal gyrus<br>- Pars opercularis, pars triangularis, and pars orbitalis<br>- Pericalcarine<br>- Postcentral gyrus<br>- Precentral gyrus<br>- Precuneus<br>- Rostral and caudal middle frontal gyrus<br>- Rostral anterior, caudal anterior, posterior and isthmus of the cingulate<br>- Superior, middle and inferior temporal gyrus<br>- Supramarginal gyrus<br>- Temporal pole<br>- Transverse temporal gyrus
**Subcortical Volumes**	Volumes (mm^3):<br>- Bilateral cerebellum<br>- Bilateral thalamus<br>- Bilateral caudate<br>- Bilateral putamen<br>- Bilateral pallidum<br>- Bilateral hippocampus<br>- Bilateral amygdala<br>- Bilateral accumbens<br>- Bilateral ventral DC<br>- Brainstem<br>- CC_Posterior<br>- CC_Mid_Posterior<br>- CC_Central<br>- CC_Mid_Anterior<br>- CC_Anterior
**Clinical assessments**	- RBDQ-HK-factor1<br>- RBDQ-HK-factor2<br>- HADS-D<br>- HADS-A<br>- MoCA (10 items)<br>- UPDRS-III (27items)

Abbreviations: CC, cingulate cortex; RBDQ-HK, Rapid eye movement sleep behavior disorder questionnaire-Hong Kong; HADS-D/A, Hospital Anxiety and Depression Scale-Depression/Anxiety score; MoCA, Montreal Cognitive Assessment; UPDRS, Unified Parkinson's Disease Rating Scale.

Table S2. Clinical and neurogenerative markers of iRBD biotypes and controls.

	Biotype 1[1]	Biotype 2[2]	Control[3]	*p* value[&]	Post-hoc[@]
TIV, mm^3, mean (SD)	1.51 (0.13) $\times 10^6$	1.50 (0.13) $\times 10^6$	1.46 (0.15) $\times 10^6$	0.016	1=2, 1>3, 2=3
Handedness, left (%)	2 (2.3%)	2 (3.5%)	3 (2.4%)	0.86	-
MoCA-HK, subscale score, mean (SD)					
Executive and visuospatial function	4.00 (0.87)	4.28 (0.85)	4.27 (0.76)	0.03	-
Naming	2.89 (0.34)	2.94 (0.23)	2.87 (0.35)	0.36	-
Attention	5.29 (0.88)	5.51 (0.98)	5.41 (1.19)	0.39	-
Language	2.93 (0.26)	2.92 (0.38)	2.86 (0.56)	0.51	-
Abstraction	1.23 (0.76)	1.34 (0.78)	1.37 (0.68)	0.40	-
Delayed recall	2.76 (1.49)	3.21 (1.6)	3.56 (1.23)	<0.001	1=2, 1<3, 2=3
Orientation	5.89 (0.34)	5.84 (0.36)	5.92 (0.28)	0.30	-
UPDRS-III, subscale score, mean (SD)					
Tremor	1.16 (1.41)	0.67 (1.20)	0.23 (0.70)	<0.001	1>2>3
Rigidity	1.24 (2.42)	0.60 (1.46)	0.10 (0.40)	<0.001	1>2=3
Bradykinesia	3.51 (3.66)	1.59 (2.88)	0.82 (1.80)	<0.001	1>2=3
HADS, subscale score, mean (SD)					
Depression score	5.60 (3.69)	5.91 (3.86)	4.16 (3.25)	0.007	1=2>3
Anxiety score	4.87 (3.63)	5.87 (3.90)	3.68 (3.16)	0.002	1=2>3
**Neurodegenerative risk factors and biomarkers**					
Regular pesticide exposure, n (%)	1 (1.56)	2 (2.86)	4 (4.88)	0.52	-
Nonuse of caffeine n (%)	34 (43.59)	32 (39.02)	41 (37.27)	0.68	-
Nonsmoking, n (%)	53 (62.35)	68 (79.07)	113 (90.4)	<0.001	1<2=3
First-degree relatives with neurogenerative disease, n (%)					
PD	5 (6.49)	9 (11.69)	0	0.002	1=2>3
Dementia	8 (10.39)	14 (17.95)	0	<0.001	1=2>3

Diabetes mellitus (type II), n (%)	8 (13.79)	8 (14.04)	14 (19.44)	0.61	-
Subthreshold parkinsonism, n (%)	51 (59.3)	26 (30.59)	19 (15.57)	<0.001	1>2>3
Olfactory loss, n (%)	56 (72.73)	48 (61.54)	17 (14.91)	<0.001	1=2>3
Constipation, n (%)	43 (51.19)	34 (40.48)	7 (6.03)	<0.001	1=2>3
Erectile dysfunction, Male only, n (%)	22 (42.31)	12 (24.49)	7 (13.46)	0.004	1=2, 1>3, 2=3
Urinary dysfunction, n (%)	6 (7.5)	8 (10)	6 (5.77)	0.56	-
Orthostatic Blood Pressure drop, mmHg, mean (SD)					
Systolic Blood Pressure drop	5.46 (14.27)	0.75 (13.36)	-2.45 (10.92)	0.001	1=2, 1>3, 2=3
Diastolic Blood Pressure drop	-0.57 (8.83)	-2.28 (7.27)	-4.49 (7.48)	0.007	1=2, 1>3, 2=3
**Physical activity**					
Strenuous exercise, hrs per week	90.13 (294.53)	50.96 (135.64)	60.16 (165.59)	0.43	-
Moderate exercise, hrs per week	115.83 (168.02)	140.18 (253.65)	121.37 (187.09)	0.72	-
Mild exercise, hrs per week	336.68 (427.15)	355.52 (514.48)	294.58 (391.80)	0.59	-
**Long-term (3-months) medications history**					
Melatonin only, n (%)	6 (11.53)	4 (7.27)	-	0.45	-
Clonazepam only, n (%)	15 (28.85)	14 (25.45)	-	0.69	-
Melatonin + Clonazepam, n (%)	12 (23.08)	6 (10.91)	-	0.09	

[&]*p* value was calculated with one-way ANOVA, ANCOVA or chi-square test.

[@]Bonferroni correction was applied to adjust for multiple comparisons of post-hoc analyses, significance was thresholded at $p < 0.017$.

Abbreviation: iRBD, isolated Rapid eye movement sleep behavior disorder; TIV, total incranial volume; MoCA, Montreal Cognitive Assessment; UPDRS, Unified Parkinson's Disease Rating Scale; HADS, Hospital Anxiety and Depression Scale; SCOPA-AUT, Scales for Outcomes in Parkinson's Disease-Autonomic; OIT, Olfactory Identification Test; MDS; Movement Disorder Society; LR, Likelihood ratio; PD, Parkinson’s disease.

Table S3. Cortical thickness of cortical regions in Biotype 1, Biotype 2 and control.

	Left hemisphere (mean, mm)					Right hemisphere (mean, mm)				
Label	Biotype 1	Biotype 2	Control	*p* value&	Post-hoc@	Biotype 1	Biotype 2	Control	*p* value&	Post-hoc@
Banks STS	2.37	2.35	2.37	0.418	-	2.44	2.35	2.45	0.47	-
Caudal anterior cingulate	2.48	2.42	2.46	0.274	-	2.35	2.42	2.35	0.609	-
Caudal middle frontal	2.53	2.51	2.53	0.292	-	2.50	2.51	2.50	0.532	-
Cuneus	1.74	1.73	1.72	0.691	-	1.82	1.73	1.81	0.349	-
Entorhinal	3.20	3.23	3.27	0.416	-	3.35	3.23	3.37	0.949	-
Fusiform	2.63	2.63	2.64	0.953	-	2.65	2.63	2.65	0.882	-
Inferior parietal	2.32	2.34	2.36	0.737	-	2.35	2.34	2.37	0.823	-
Inferior temporal	2.71	2.71	2.74	0.222	-	2.73	2.71	2.78	0.045	-
Isthmus cingulate	2.22	2.20	2.24	0.099	-	2.19	2.20	2.22	0.149	-
Lateral occipital	2.07	2.06	2.06	0.407	-	2.12	2.06	2.12	0.63	-
Lateral orbitofrontal	2.56	2.52	2.55	0.053	-	2.61	2.52	2.59	0.13	-
Lingual	1.84	1.83	1.83	0.916	-	1.88	1.83	1.87	0.813	-
Medial orbitofrontal	2.40	2.38	2.41	0.172	-	2.43	2.38	2.45	0.003	-
Middle temporal	2.73	2.73	2.76	0.218	-	2.74	2.73	2.77	0.517	-
Parahippocampal	2.43	2.47	2.44	0.526	-	2.39	2.47	2.44	0.337	-
Paracentral	2.43	2.43	2.41	0.203	-	2.46	2.43	2.46	0.186	-
Pars opercularis	2.51	2.50	2.51	0.425	-	2.53	2.50	2.52	0.504	-
Pars orbitalis	2.64	2.61	2.63	0.444	-	2.67	2.61	2.66	0.303	-
Pars triangularis	2.35	2.36	2.37	0.944	-	2.40	2.36	2.39	0.175	-
Pericalcarine	1.50	1.46	1.46	0.036	-	1.54	1.46	1.51	0.11	-
Postcentral	2.03	2.03	2.04	0.469	-	2.03	2.03	2.03	0.68	-
Posterior cingulate	2.37	2.33	2.37	0.097	-	2.35	2.33	2.36	0.932	-
Precentral	2.52	2.54	2.53	0.81	-	2.45	2.54	2.46	0.062	-
Precuneus	2.25	2.27	2.28	0.761	-	2.27	2.27	2.29	0.76	-
Rostral anterior cingulate	2.66	2.64	2.68	0.494	-	2.71	2.64	2.68	0.784	-
Rostral middle frontal	2.37	2.34	2.36	0.188	-	2.39	2.34	2.37	0.161	-
Superior frontal	2.72	2.70	2.72	0.271	-	2.70	2.70	2.71	0.416	-
Superior parietal	2.12	2.12	2.13	0.726	-	2.08	2.12	2.10	0.74	-
Superior temporal	2.59	2.61	2.63	0.786	-	2.63	2.61	2.66	0.932	-
Supramarginal	2.42	2.44	2.44	0.761	-	2.41	2.44	2.44	0.969	-
Frontal pole	2.74	2.69	2.72	0.189	-	2.75	2.69	2.75	0.153	-
Temporal pole	3.50	3.52	3.55	0.854	-	3.57	3.52	3.65	0.18	-
Transverse temporal	2.25	2.21	2.22	0.245	-	2.26	2.21	2.25	0.307	-
Insula	2.87	2.87	2.87	0.948	-	2.88	2.87	2.92	0.229	-

&*p* value was calculated with one-way ANOVA, and Bonferroni correction was applied to adjust for multiple comparisons of 34 regions on each hemisphere, a $p \leqslant 0.001$ is considered statistical significance.

@Bonferroni correction was applied to adjust for multiple comparisons of three groups, significance was thresholded at $p < 0.017$.

Table S4. Surface area of cortical regions in Biotype1, Biotype 2 and control.

	Left hemisphere (mean, mm^2)					Right hemisphere (mean, mm^2)				
Label	Biotype1	Biotype2	Control	p value&	Post-hoc@	Biotype1	Biotype2	Control	p value&	Post-hoc@
Banks STS	879.10	917.43	890.74	0.107	-	773.74	814.66	798.80	0.033	-
Caudal anterior cingulate	541.45	554.43	550.82	0.412	-	669.35	720.05	675.90	0.021	-
Caudal middle frontal	1877.47	2013.90	1994.77	<0.001	1<2=3	1764.30	1870.71	1877.60	<0.001	1<2=3
Cuneus	1443.45	1543.23	1487.89	0.001	1<2=3	1530.43	1615.73	1546.03	0.006	-
Entorhinal	391.71	436.17	401.74	<0.001	1=3<2	381.23	391.79	362.14	0.103	-
Fusiform	2876.81	3032.31	2870.10	<0.001	1<3<2	2853.74	2941.51	2796.88	0.045	-
Inferior parietal	4098.60	4345.98	4231.19	<0.001	1<2=3	4797.20	5195.31	4862.88	<0.001	1<3<2
Inferior temporal	3113.24	3348.28	3184.90	<0.001	1<2=3	2971.57	3184.88	3044.48	<0.001	1<2=3
Isthmus cingulate	967.93	1033.00	971.29	<0.001	1<2=3	890.35	942.85	891.56	0.002	-
Lateral occipital	4590.66	4883.20	4692.66	<0.001	1<2=3	4616.80	4786.56	4607.65	0.053	-
Lateral orbitofrontal	2376.78	2577.58	2461.61	<0.001	1<2=3	2315.06	2549.14	2416.90	<0.001	1<3<2
Lingual	2830.63	3026.23	2859.44	<0.001	1=3<2	2952.02	3167.60	3013.88	0.001	1<2=3
Medial orbitofrontal	1854.35	1904.28	1857.69	0.018	-	1912.85	1999.00	1924.56	<0.001	1<3<2
Middle temporal	2867.12	3039.65	2972.65	<0.001	1<2=3	3119.69	3348.03	3225.53	<0.001	1<2=3
Parahippocampal	613.21	648.30	618.94	0.001	1=3<2	595.81	618.15	609.94	0.02	-
Paracentral	1225.98	1255.05	1245.10	0.196	-	1308.95	1362.49	1341.26	0.04	-
Pars opercularis	1401.65	1488.40	1449.48	0.012	-	1222.05	1269.52	1262.10	0.028	-
Pars orbitalis	601.69	639.08	609.07	<0.001	1<2=3	711.41	762.37	720.30	<0.001	1<3<2
Pars triangularis	1174.83	1259.99	1197.48	0.001	1<2=3	1429.27	1487.22	1440.67	0.166	-
Pericalcarine	1355.95	1443.16	1369.38	0.047	-	1511.52	1621.70	1527.90	0.004	-
Postcentral	3830.70	3999.30	3899.47	0.004	-	3729.31	3874.00	3793.81	0.03	-
Posterior cingulate	1060.48	1131.97	1080.63	0.007	-	1078.41	1164.84	1117.26	0.001	1<2=3
Precentral	4467.59	4678.91	4578.92	<0.001	1<2=3	4389.45	4616.70	4507.60	<0.001	1<2=3
Precuneus	3510.66	3739.06	3508.24	<0.001	1<2=3	3651.23	3865.98	3685.08	<0.001	1<2=3
Rostral anterior cingulate	745.15	815.70	760.69	0.002	-	557.38	613.97	577.36	0.002	-
Rostral middle frontal	4897.29	5286.43	5073.70	<0.001	1<2=3	4927.78	5169.80	5055.21	0.023	-
Superior frontal	6327.30	6660.35	6498.40	<0.001	1<2=3	6044.13	6162.24	6086.99	0.058	-
Superior parietal	4956.66	5238.93	5020.42	0.003	-	4777.99	5053.44	4875.15	0.004	-
Superior temporal	3622.98	3777.34	3709.77	<0.001	1<2=3	3376.80	3540.07	3467.85	<0.001	1<2=3
Supramarginal	3704.23	3938.31	3818.68	0.005	-	3275.37	3488.20	3417.71	<0.001	1<2=3
Frontal pole	253.08	256.38	255.97	0.087	-	316.33	321.23	312.53	0.381	-
Temporal pole	506.94	533.50	509.17	0.008	-	508.45	511.84	502.14	0.612	-
Transverse temporal	429.15	444.02	440.12	0.022	-	306.05	324.20	317.90	<0.001	1<2=3
Insula	2253.14	2365.17	2272.75	<0.001	1<2=3	2195.83	2269.87	2199.78	0.002	-

&*p* value was calculated with one-way ANOVA, and Bonferroni correction was applied to adjust for multiple comparisons of 34 regions on each hemisphere, a $p \leqslant 0.001$ is considered statistical significance.

@Bonferroni correction was applied to adjust for multiple comparisons of three groups, significance was thresholded at $p < 0.017$.

Table S5. Volume of cortical regions in Biotype1, Biotype 2 and control.

	Left hemisphere (mean, mm^3)					Right hemisphere (mean, mm^3)				
Label	Biotype1	Biotype2	Control	p value[&]	Post-hoc[@]	Biotype1	Biotype2	Control	p value[&]	Post-hoc[@]
Banks STS	1986.73	2072.01	2027.79	0.259	-	1792.35	1917.70	1861.65	0.022	-
Caudal anterior cingulate	1427.79	1448.16	1471.18	0.453	-	1759.62	1929.98	1799.21	0.009	-
Caudal middle frontal	5264.38	5533.88	5529.74	0.003	-	4884.99	5133.00	5194.21	<0.001	-
Cuneus	2723.38	2839.52	2716.13	0.166	-	3060.56	3176.23	3051.67	0.266	-
Entorhinal	1826.00	2024.17	1909.63	<0.001	1<2=3	1895.62	1930.41	1805.83	0.233	-
Fusiform	8762.29	9163.97	8720.21	0.013	-	8757.05	8965.13	8479.11	0.164	-
Inferior parietal	10385.94	11019.69	10876.10	0.001	1<2=3	12386.43	13469.41	12644.38	<0.001	1=3<2
Inferior temporal	10069.09	10717.84	10337.30	0.001	1<2=3	9673.28	10230.19	10031.11	0.001	1<2=3
Isthmus cingulate	2393.91	2524.66	2417.37	0.006	-	2203.90	2319.58	2235.11	0.008	-
Lateral occipital	10619.23	11085.40	10714.88	0.037	-	11024.98	11238.23	10974.85	0.448	-
Lateral orbitofrontal	6486.95	6878.52	6643.56	<0.001	1<2=3	6417.26	6906.35	6622.44	<0.001	1<2=3
Lingual	5611.17	5972.42	5634.41	0.01	-	6072.19	6383.17	6115.43	0.046	-
Medial orbitofrontal	4853.90	4915.67	4895.13	0.039	-	5174.86	5265.71	5192.26	0.062	-
Middle temporal	9554.16	10009.01	10009.18	<0.001	1<2=3	10329.06	11056.80	10779.44	<0.001	1<2=3
Parahippocampal	1745.81	1879.90	1781.15	0.004	-	1669.55	1770.48	1752.66	0.007	-
Paracentral	3278.36	3347.36	3282.74	0.766	-	3509.20	3660.30	3576.09	0.201	-
Pars opercularis	3942.00	4110.38	4053.45	0.094	-	3401.93	3513.36	3494.25	0.14	-
Pars orbitalis	2001.37	2086.06	1998.54	0.071	-	2377.09	2479.02	2390.64	0.066	-
Pars triangularis	3099.15	3297.55	3169.33	0.009	-	3904.60	3969.70	3882.29	0.765	-
Pericalcarine	1835.48	1886.55	1787.12	0.486	-	2157.26	2241.17	2121.86	0.282	-
Postcentral	8699.95	9033.50	8862.01	0.11	-	8399.40	8746.30	8540.02	0.095	-
Posterior cingulate	2712.92	2844.12	2753.37	0.113	-	2755.38	2972.34	2871.10	0.004	-
Precentral	12296.17	12940.34	12661.40	0.004	-	11689.42	12531.71	11997.38	0.001	1<2, 1=3, 2=3
Precuneus	8366.12	8938.00	8495.85	<0.001	1<2=3	8724.03	9297.37	8909.61	<0.001	1<2=3
Rostral anterior cingulate	2224.13	2417.66	2288.66	0.004	-	1757.93	1928.44	1799.18	0.005	-
Rostral middle frontal	13260.86	13833.02	13442.24	0.031	-	13879.69	14071.92	13874.70	0.326	-
Superior frontal	19962.72	20485.09	20175.75	0.033	-	18980.40	19006.71	18921.56	0.26	-
Superior parietal	11551.72	12125.55	11767.71	0.053	-	10811.40	11616.98	11246.59	0.003	-
Superior temporal	10738.07	11179.49	11080.79	0.008	-	10180.07	10698.30	10522.06	<0.001	1<2=3
Supramarginal	9838.98	10466.24	10212.37	0.01	-	8579.42	9195.12	9022.43	<0.001	1<2=3
Frontal pole	945.65	905.81	921.67	0.215	-	1186.52	1140.31	1153.13	0.382	-
Temporal pole	2601.36	2706.47	2669.75	0.129	-	2614.50	2727.85	2677.60	0.064	-
Transverse temporal	1046.10	1066.41	1046.65	0.759	-	767.13	797.64	786.07	0.2	-
Insula	6456.78	6725.28	6511.17	<0.001	1<2=3	6345.74	6531.91	6408.29	0.003	-

[&]*p* value was calculated with one-way ANOVA, and Bonferroni correction was applied to adjust for multiple comparisons of 34 regions on each hemisphere, a $p \leqslant 0.001$ is considered statistical significance.

[@]Bonferroni correction was applied to adjust for multiple comparisons of three groups, significance was thresholded at $p < 0.017$.

Table S6. Subcortical volume (mean, mm^3) in Biotype 1, Biotype 2 and control.

	Biotype 1	Biotype 2	Control	*p* value	Post-hoc[@]
L-Cerebellum	49558.52	52350.40	51185.33	<0.001[&]	1<2=3
L-Thalamus	6804.39	7350.91	7188.58	<0.001[&]	1<2=3
L-Caudate	3007.83	3244.87	3187.05	<0.001[&]	1<2=3
L-Putamen	4170.92	4566.53	4448.42	<0.001[&]	1<2=3
L-Pallidum	1910.28	2022.44	1958.67	0.001[&]	1<2=3
Brainstem	20208.94	21332.11	20884.29	<0.001[&]	1<2=3
L-Hippocampus	3691.23	3999.65	3951.14	<0.001[&]	1<2=3
L-Amygdala	1415.79	1537.26	1523.78	<0.001[&]	1<2=3
L-Accumbens	287.13	334.53	334.40	<0.001[&]	1<2=3
L-Ventral DC	3829.08	4033.39	3949.12	<0.001[&]	1<2=3
R-Cerebellum	50811.37	52876.30	51877.91	0.007[&]	-
R-Thalamus	6878.99	7459.46	7218.10	<0.001[&]	1<2=3
R-Caudate	3033.63	3257.44	3183.87	0.001[&]	1<2=3
R-Putamen	4256.54	4607.26	4493.26	<0.001[&]	1<2=3
R-Pallidum	1882.49	1961.79	1929.42	0.023[&]	-
R-Hippocampus	3869.60	4178.17	4128.86	<0.001[&]	1<2=3
R-Amygdala	1633.25	1721.84	1710.33	<0.001[&]	1<2=3
R-Accumbens	397.59	449.82	445.73	<0.001[&]	1<2=3
R-Ventral DC	3735.59	3990.02	3901.87	<0.001[&]	1<2=3
CC_Posterior	972.13	1021.63	1019.60	0.022[%]	-
CC_Mid_Posterior	501.39	540.11	516.49	0.134[%]	-
CC_Central	504.99	579.43	567.65	0.003[%]	1<2=3
CC_Mid_Anterior	469.43	565.43	546.49	<0.001[%]	1<2=3
CC_Anterior	812.10	851.36	856.28	0.013[%]	-

[&] *p* value was calculated with one-way ANOVA, and Bonferroni correction was applied to adjust for multiple comparisons of 10 regions on each hemisphere, a p<0.005 is considered statistical significance.

[%] *p* value was calculated with one-way ANOVA, and Bonferroni correction was applied to adjust for multiple comparisons of 5 regions, a p<0.01 is considered statistical significance.

[@]Bonferroni correction was applied to adjust for multiple comparisons of three groups, significance was thresholded at p < 0.017.

Abbreviations: L, left; R, right; CC, cingulate cortex.

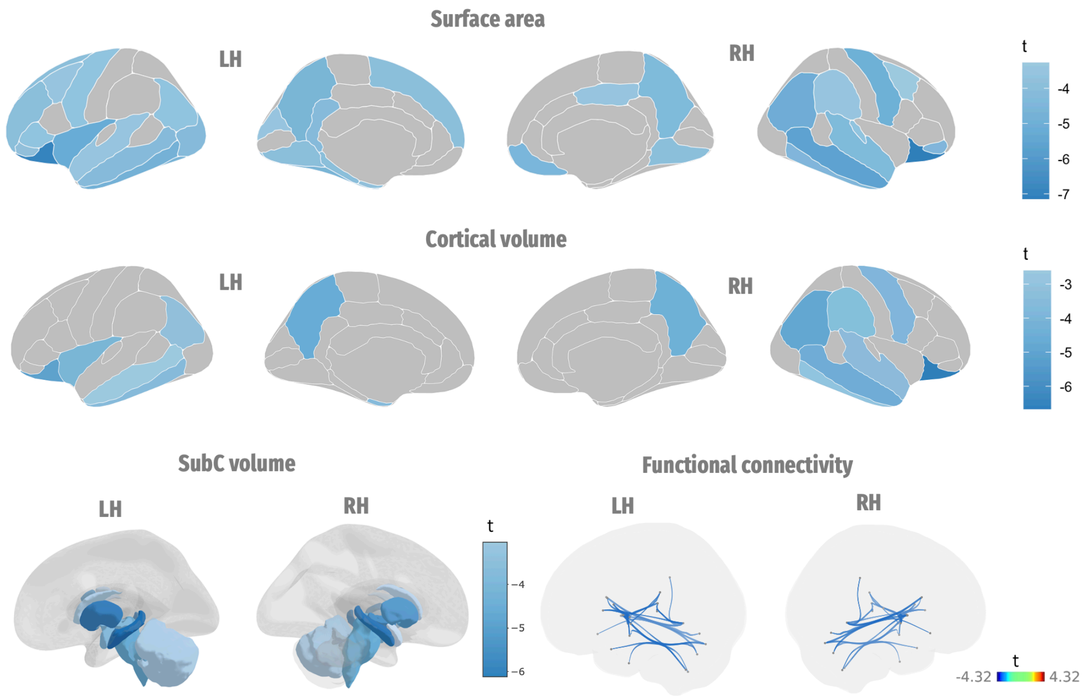

Fig. S1. Group differences of cortical surface area, volume, subcortical volume, and functional connectivity between Biotype 1 and Biotype 2 patients. The color bar represents t-statistics, with red indicating increase and blue indicating decrease. L/RH, left/right hemisphere.

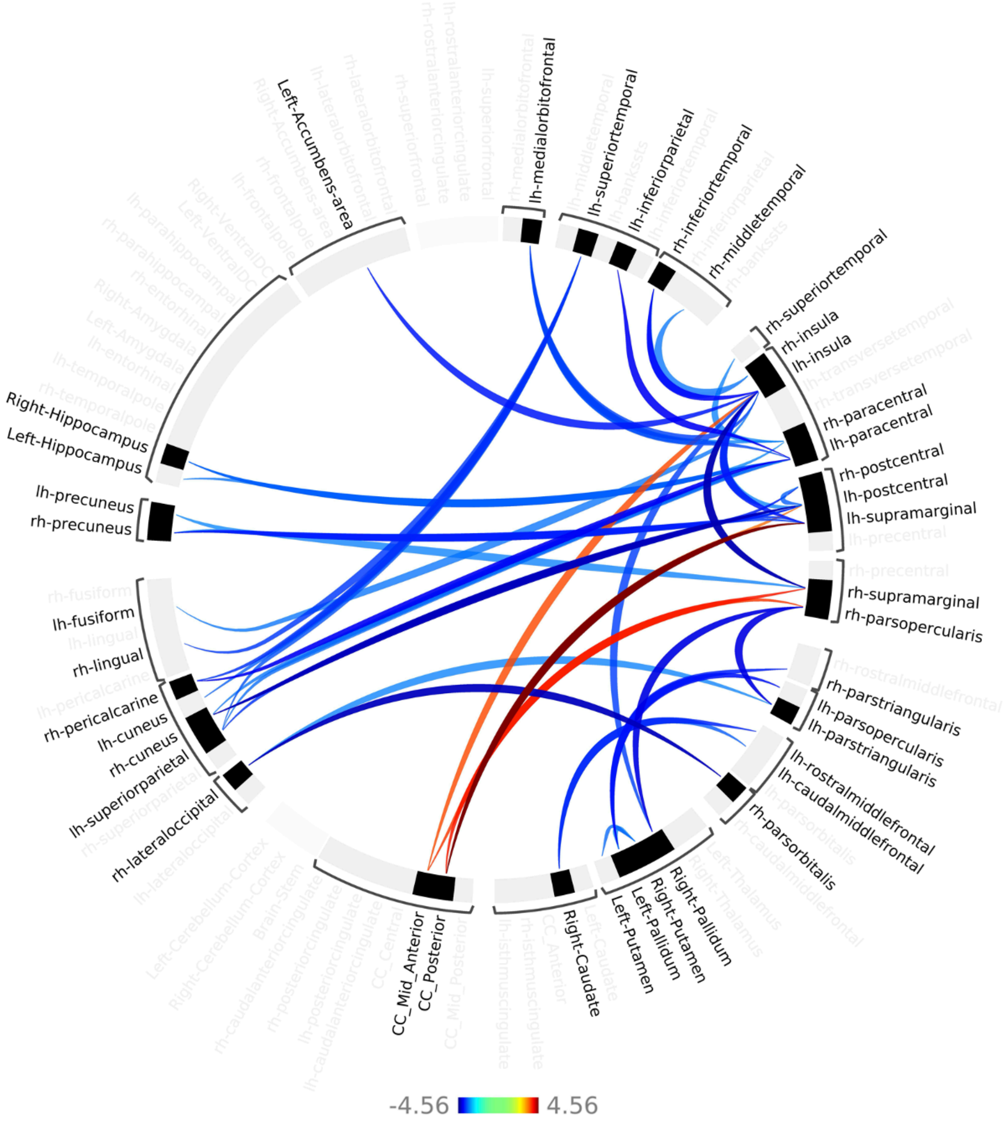

Fig. S2. Functional connectivity differences between Biotype 1 and the control group using Desikan-Killiany and aseg atlas. Only significant connections are displayed (thresholded at connection level $p<0.001$ and multiple-comparison corrected using p-FDR<0.05). The color bar represents t-statistics, with warm colors indicating hyperconnectivity and cold colors indicating hypoconnectivity.
